# Clinical correlations and prognostic value of Nudix hydroxylase 10 in patients with gastric cancer

**DOI:** 10.1080/21655979.2021.1995104

**Published:** 2021-12-02

**Authors:** Diqun Chen, Rouxin Zhang, Aosi Xie, Jinpeng Yuan, Jinhai Zhang, Yongjian Huang, Hongxia Zhang, Feiran Zhang

**Affiliations:** a Department of Gastrointestinal Surgery, The First Affiliated Hospital of Shantou University Medical College, Shantou, China; b College of Science and Technology, China Three Gorges University, Yichang, China; c Department of Abdominal Surgery, Cancer Hospital of Shantou University Medical College, Shantou, China; d Department of Gastrointestinal Surgery, Shantou Guorui Hospital, Shantou, China; e Health Care Center, The First Affiliated Hospital of Shantou University Medical College, Shantou, China

**Keywords:** NUDT10, gastric cancer, biomarker, independent predictor, prognosis

## Abstract

Gastric cancer (GC) is one of the most common and lethal cancers worldwide. The Nudix hydroxylase (NUDT) genes have been reported to play notable roles in tumor progression. However, the role of NUDT10 in GC has not been reported. In this study, we investigated the expression of NUDT10 in GC and its association with clinicopathological characteristics. Quantitative real-time polymerase chain reaction and analyses of The Cancer Genome Atlas and Human Protein Atlas databases were performed to determine NUDT10 mRNA and protein expression. Receiver operating characteristic curve analysis was used to assess the diagnostic value of NUDT10 in patients with GC. We used Cox regression and the Kaplan–Meier method to assess the correlations between clinicopathological factors and survival outcomes of patients with GC. Gene set enrichment analysis (GSEA) was performed to identify the underlying signaling pathways. NUDT10 mRNA and protein expression was significantly lower in GC tissues compared to normal tissues. Interestingly, higher NUDT10 expression was correlated with advanced tumor stage, deeper local invasion, and worse survival outcomes. Patients with higher NUDT10 expression had a significantly worse prognosis than those with lower NUDT10 expression. Multivariate analysis showed that high NUDT10 expression was an independent predictor of survival outcome. Several pathways, including mismatch repair, nucleotide excision repair, extracellular matrix receptor interaction, and cancer signaling, were identified as enriched pathways in GC through GSEA. To our knowledge, this study is the first to characterize NUDT10 expression in GC. Our study demonstrates that NUDT10 is a promising independent biomarker for GC prognosis.

## Introduction

Gastric cancer (GC) is the fifth most common neoplasm and third leading cause of cancer-related deaths worldwide, and over a million new cases of GC are diagnosed each year [1]. Although its incidence has steadily declined over the past 50 years, the five-year overall survival rate of GC remains low due to the delay in diagnosis [2]. GC is highly aggressive and typically asymptomatic, and the majority of patients with GC are diagnosed at an advanced stage and with distant metastasis [3]. Therefore, novel effective biomarkers are urgently required for the early detection and precise prognosis of patients with GC.

Nudix hydroxylases (NUDTs) are a family of Mg^2+^-requiring enzymes found in all classes of organisms that catalyze the hydrolysis of a wide range of nucleoside pyrophosphates linked to other moieties of amino acids [4]. All NUDTs consist of a Nudix hydroxylase fold and Nudix box, which is a conserved 23-residue sequence motif(GXXXXXEXXXXXXXREUXEEXGU, where U is a hydrophobic residue and X is any amino acid) [5]. During the process of eliminating hydrolytic substrates, NUDT plays a signaling and regulatory role in metabolism [6]. NUDT members have been reported to participate in the development and progression of several malignancies, including leukemia, renal, breast, and prostate cancers, which are associated with adverse outcomes [7–10]. Several genome-wide association studies have indicated that NUDT10, a member of the NUDT family located in Xp11.22, is associated with overall survival in prostate cancer [11–13]. A recent study has implicated that low expression of NUDT10 can increase promoter methylation in prostate cancer, exhibiting a tumor suppressor characteristic [14]. However, the specific role of NUDT10 in GC remains unknown.

Considering the roles of NUDT family members in tumor progression reported in previous studies, we speculate that NUDT10 might have potential oncogenic peculiarity in GC. In this study, we aimed to explore the clinicopathological significance and prognostic value, as well as the underlying molecular signaling pathways of NUDT10 in GC.

## Materials and methods

### Tumor samples

GC and corresponding adjacent nontumor tissues (50 pairs) were collected from patients who underwent surgery at the First Affiliated Hospital of Shantou University Medical College between 2019 and 2020. All specimens were immediately frozen after surgery and stored at −80°C. This study was approved by the Institutional Research Ethics Committee of the First Affiliated Hospital of Shantou University Medical College. All patients who participated in this study provided written informed consent before surgery.

### Data mining

The gene expression quantification (workflow type: high-throughput sequencing [HTSeq]-Counts; 375 cancer and 32 normal samples included) and corresponding clinical data with survival time of patients with GC were obtained from the Genomic Data Commons data portal of The Cancer Genome Atlas (TCGA; https://portal.gdc.cancer.gov/repository; public data updated until 7 April 2020). Boxplots were used to visualize the distribution of the discrete clinical variates. Using this HTSeq-Count data of the gene expression of 375 patients with GC, we analyzed the correlation between the NUDT10 expression level and the clinical factors and survival outcomes for patients with GC. The Human Protein Atlas (HPA; https://www.proteinatlas.org/) project contains an expression map of the complete human proteome in normal and cancerous tissues with distribution information of more than 20,000 human proteins [15]. Further validation of the protein expression difference was conducted through the analysis of immunohistochemistry images obtained from this database. The Kaplan–Meier Plotter database (http://kmplot.com/), which summarizes the gene expression and survival correlation of various cancer types [16], including gastric cancer (https://kmplot.com/analysis/index.php?p=service&cancer=gastric), was used to verify the prognostic ability of NUDT10.

### Quantitative real-time polymerase chain (qPCR) reaction

RNA was extracted from the tissues using TRIzol reagent (Thermo Fisher Scientific, Waltham, MA, USA) and reverse transcribed to cDNA using the Geneseed® II First Strand cDNA Synthesis Kit (Geneseed, Guangzhou, China). The primers used for NUDT10 amplification were as follows: forward, 5ʹ-GACAGGTGAGCTCTTTCACACTC-3ʹ;reverse,5ʹ-GGAGTTATG TCTAGAGGCACAGTC-3ʹ. For qPCR, a 20-µL reaction containing 10 µL 2x qPCR SYBR-Green 30 master mix (Vazyme Biotech, Nanjing, China), 0.4 µL forward primer (10 µM), 0.4 µL reverse primer (10 µM), and 5 µL cDNA were included in the 20 µL reaction system. All specimens were tested in triplicate. Relative mRNA levels of *NUDT10* were normalized to those of *GAPDH*.

### Statistical analyses

Perl Programming Language (v5.30.0) and R (v3.6.3) software were used for data preparation and analysis. The Wilcoxon rank-sum test in the ’limma’ R package [17] was used to analyze differentially expressed genes in both the TCGA and validation cohorts between normal and GC tissues. In addition, the relationships between NUDT10 expression and clinicopathologic parameters were evaluated in the TCGA and validation cohorts using the Chi-square test and logistic regression [18]. The receiver operating characteristic (ROC) curve is a method used to assess the discrimination accuracy of a diagnostic test over the range of possible cutoff points for the predictor variable [19]. The ROC curve was used to evaluate the diagnostic value of NUDT10 for GC. The Kaplan–Meier method and Cox regression analysis were used to evaluate the prognostic value of NUDT10. Statistical significance was set at *P*< 0.05.

### Gene set enrichment analysis (GSEA)

GSEA is a method used to distinguish differential expression of gene sets between subgroups and to explore potential molecular signaling pathways [20]. The phenotype labels of NUDT10 expression data (375 tumor samples) extracted from TCGA were divided into high and low NUDT10 subgroups based on the median values. The phenotype label files and datasets were uploaded to GSEA software. Each analysis was conducted 1000 times for the gene set permutations. Gene sets were defined as enriched only when both the normal *P*-value and false discovery rate (FDR) q-values were less than 0.05.

## Results

Considering the roles of the NUDT family and NUDT10 in tumor progression reported in previous studies, we hypothesized that NUDT10 might play an important role in the occurrence and development of GC. In this study, we investigated the expression of NUDT10 in GC and its clinical relevance using the TCGA dataset and our own validation cohort. We aimed to explore the clinicopathologic significance, prognostic value, and underlying signaling pathways of NUDT10 in GC.

### NUDT10 is downregulated in gastric cancer

**Figure 1.**
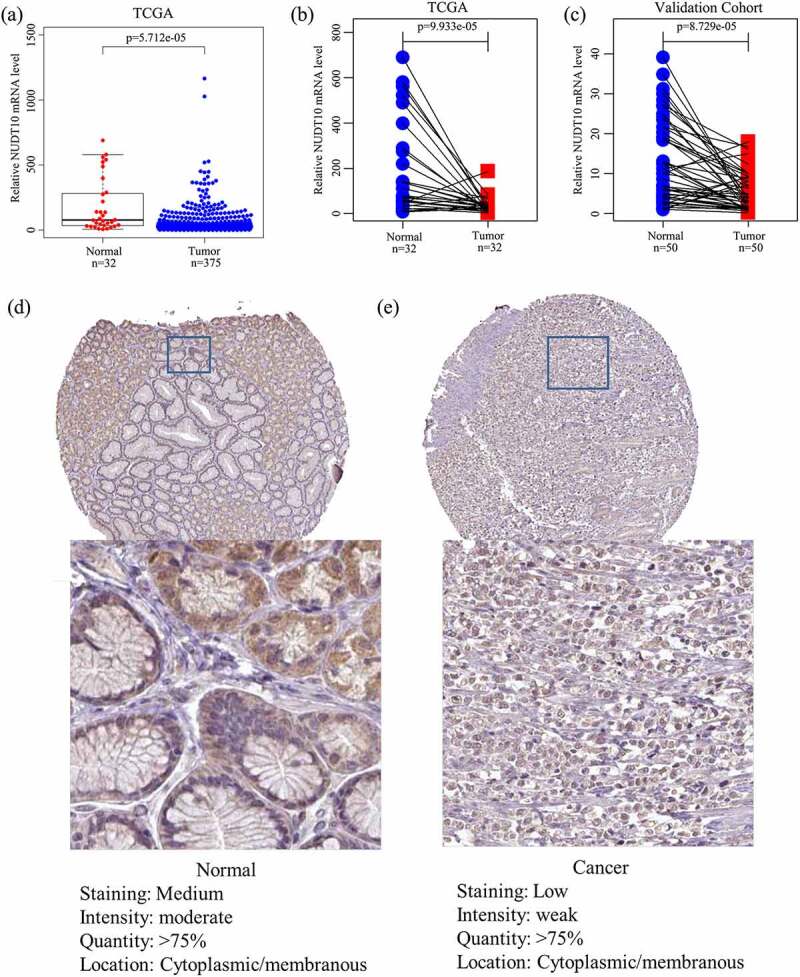
NUDT10 expression in gastric cancer (GC) and adjacent normal tissues. (a-c): The TCGA and validation cohorts indicate that NUDT10 is downregulated in GC. (d-e): Validation of NUDT10 protein expression level via cell immunohistochemistry of normal and GC tissues using the Human Protein Atlas database

A total of 407 samples (375 tumor and 32 adjacent nontumor tissues) with corresponding clinical data were identified in the TCGA cohort. Baseline features are shown in Table 1. We determined NUDT10 expression in tumor and adjacent normal samples and paired samples in the TCGA and validation cohorts. The results revealed that *NUDT10* expression was significantly lower in GC tissues than in normal tissues (Figure 1(a-c)). To further validate this result at the protein level, we extracted immunohistochemical staining data (with HPA05768 as the antibody) from the HPA database, which are presented in Table 1. A total of 29 samples, including six normal and 23 tumor samples, were obtained. All six normal gastric samples (100%) showed moderate staining with moderate intensity, while only 10 of 23 tumor samples (43.5%) showed moderate staining with moderate intensity. It can be approximately estimated that the staining of NUDT10 is higher in normal glandular cells than in GC cells (Figure 1(e)). This finding is consistent with our *NUDT10* results at the mRNA level.

**Table 1.** Baseline features of the TCGA (375 patients) and HPA (29 patients) cohorts

Clinical factors	Total	%
Age at diagnosis (year)	65.8 (35 ~ 90)	
Survival time (year)	1.4 (0 ~ 10.3)	
Survival status		
Alive	131	34.9
Death	244	65.1
Gender		
Female	134	35.7
Male	241	64.3
Tumor grade		
G1	10	2.7
G2	137	36.5
G3	219	58.4
Gx	9	2.4
TNM stage		
I	56	14.4
II	113	30.9
III	165	40.5
IV	41	10.1
T classification		
T1	19	5.1
T2	80	21.3
T3	168	44.8
T4	100	26.7
Tx	8	2.1
M classification		
M0	330	88.0
M1	25	6.7
Mx	20	5.3
N classification		
N0	112	29.9
N1	97	25.9
N2	75	20.0
N3	74	19.7
Nx	17	4.5
HPA datasets		
High or medium staining in IHC (6 normal tissue samples)	6	100
High or medium staining in IHC (23 cancer tissue samples)	10	43.5

Data are expressed as the mean (min–max); x represents unknown. Abbreviations: TCGA, The Cancer Genome Atlas; HPA, Human Protein Atlas; IHC, immunohistochemistry.

### Association of NUDT10 expression with clinicopathologic factors

In the TCGA cohort, NUDT10 expression was significantly correlated with tumor grade (*P*= 0.001), T stage (*P*< 0.001), and TNM stage (*P*= 0.002), but was not correlated with age, sex, lymph node metastasis, and distant metastasis (Table 2). Univariate logistic regression indicated that NUDT10 was correlated with some prognostic clinicopathological factors (Table 3). High NUDT10 expression in GC was correlated with T stage (odds ratio [OR] = 16.286 for T2 vs. T1; OR = 20.278 for T3 vs. T1; and OR = 22.000 for T4 vs. T1) and TNM stage (OR = 3.580 for stage II vs. stage I; OR = 2.602 for stage III vs. stage I; and OR = 2.815 for stage IV vs. stage I). Furthermore, in our validation cohort, NUDT10 expression was significantly associated with T stage (OR = 3.886, T4 vs. T1+ T2+ T3), lymph node metastasis (OR = 5.133, yes vs. no), and TNM stage (OR = 19, stage III vs. stage I + II). Other clinical parameters, such as age, sex, grade, and tumor size, were not correlated with NUDT10 expression.

**Table 2.** Association between NUDT10 expression and clinicopathologic factors in the TCGA and validation cohorts

			NUDT10 expression				
TCGA cohort		Total	Low	High	High (%)	Χ^2^	*P value*
Age	<65	155	72	83	53.5	1.433	0.231
	≥65	220	116	104	47.3		
Gender	Female	134	65	69	51.5	0.220	0.639
	Male	241	123	118	49.0		
Tumor grade	G1	10	5	5	50.0	14.535	<0.001***
	G2	137	86	51	37.2		
	G3	219	92	127	58.0		
T stage	T1	19	18	1	5.30	17.825	<0.001***
	T2	80	43	37	46.3		
	T3	168	77	91	54.2		
	T4	100	45	55	55.0		
LN metastasis	Yes	246	119	127	51.6	0.832	0.362
	No	112	60	52	46.4		
Distant metastasis	Yes	25	13	12	48.0	0.049	0.824
	No	330	164	166	50.3		
TNM stage	Stage I	56	41	15	26.8	14.575	0.002**
	Stage II	113	49	64	56.6		
	Stage III	165	78	87	52.7		
	Stage IV	41	20	21	51.2		
Validation cohort							
Age	<65	26	11	15	57.7	1.282	0.258
	≥65	24	14	10	41.7		
Gender	Female	15	9	6	40.0	0.857	0.355
	Male	35	16	19	54.2		
Tumor size	<5 cm	26	14	12	46.2	0.321	0.571
	≥5 cm	24	11	13	51.2		
Tumor grade	G1+ G2	23	13	10	43.5	0.725	0.395
	G3	27	12	15	55.6		
T stage	T1+ T2+ T3	23	8	15	65.2	3.945	0.047*
	T4	27	10	17	63.0		
LN metastasis	Yes	36	14	22	61.1	6.349	0.012*
	No	14	11	3	21.4		
TNM stage	Stage I/II	22	19	3	13.6	20.779	<0.001***
	Stage III	28	6	22	78.6		

Comparison was performed using the Chi-square test. Abbreviations: NUDT10: Nudix hydroxylase 10; LN: lymph node.

**Table 3.** Logistic regression of NUDT10 expression and clinicopathological parameters in the TCGA and validation cohorts

TCGA cohort					
Clinical features		TN	OR	95%CI	*P* value
Age	≥65 VS <65	371	0.776	0.513–1.173	0.230
Gender	Female VS Male	375	0.947	0.620–1.445	0.799
Grade	G2 VS G1	146	0.756	0.202–2.835	0.670
	G3 VS G1	228	1.190	0.323–4.391	0.788
T stage	T2 VS T1	100	16.286	3.128–299.971	0.008**
	T3 VS T1	188	20.278	4.045–368.991	0.004**
	T4 VS T1	120	22.000	4.286–403.417	0.003**
LN metastasis	Yes VS No	357	1.323	0.845–2.080	0.222
Distant metastasis	Yes VS No	355	1.097	0.483–2.507	0.824
TNM stage	stage II VS stage I	164	3.580	1.795–7.430	<0.001***
	stage III VS stage I	203	2.602	1.343–5.250	0.006**
	Stage IV VS stage I	91	2.815	1.187–6.863	0.020*
Validation cohort					
Age	≥65 VS <65	50	0.612	0.196–1.864	0.390
Gender	Female VS Male	50	1.612	0.530–5.027	0.403
Grade	G3 VS G1+ G2	50	1.944	0.635–6.185	0.249
Tumor size	≥5 cm VS <5 cm	50	1.612	0.529–5.027	0.403
T stage	T4 VS T1+ T2+ T3	50	3.886	1.229–13.342	0.025*
LN metastasis	Yes VS No	50	5.133	1.336–25.695	0.026*
TNM stage	Stage III VS Stage I/II	50	19	4.809–100.600	<0.001***

### Diagnostic value of NUDT10 in gastric cancer

**Figure 2.**
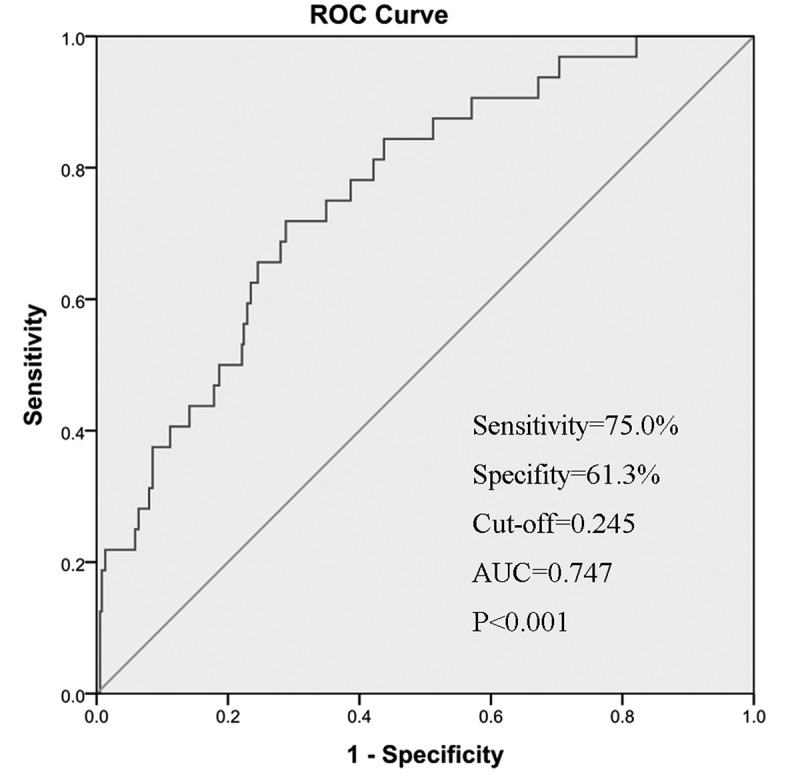
Receiver operating characteristic curve analysis for NUDT10 expression in normal and GC tissues in the TCGA cohort

The mRNA expression profiles extracted from the TCGA cohort were subjected to ROC analysis to evaluate the diagnostic accuracy of NUDT10. The area under the ROC curve was 0.761 (95% confidence interval [CI]: 66.6%-82.8%), the sensitivity was 75.0%, and the specificity was 61.3%, which shows moderate diagnostic value (Figure 2).

### Survival analysis and univariate /multivariate analyses

**Figure 3.**
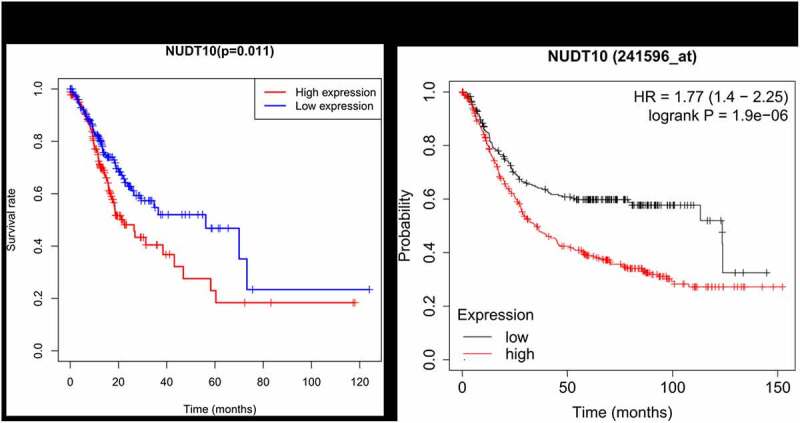
NUDT10 expression and survival analysis in the TCGA cohort (a) and Kaplan-Meier Plotter database (b)

**Figure 4.**
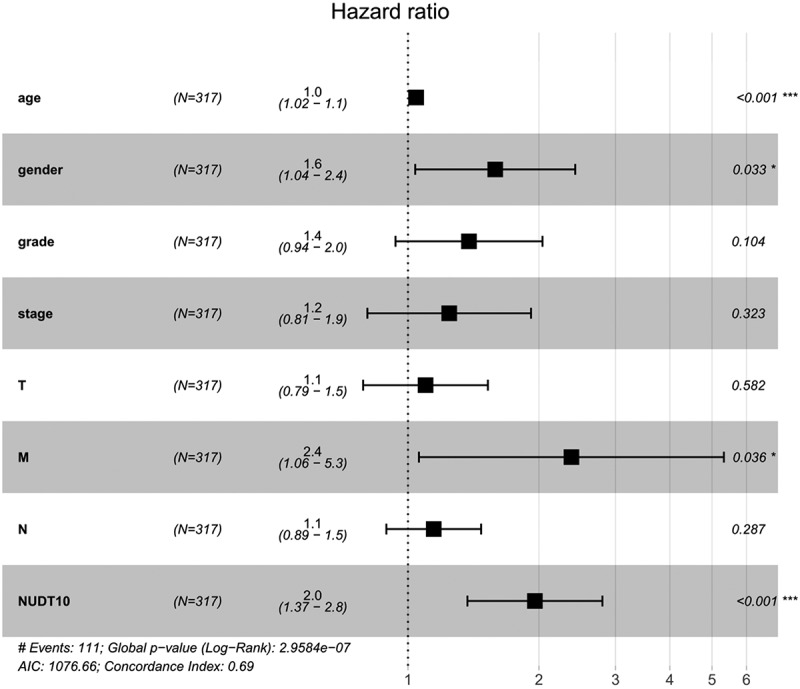
Forest map of multivariate analysis of the correlation between clinical factors and survival outcome

Patients with high NUDT10 expression were strongly correlated with worse survival outcomes (Figure 3(a), *p*= 0.011). Survival analysis of NUDT10 from the Kaplan–Meier Plotter database further confirmed this result (Figure 3(b), *p*< 0.001). As shown in Table 4, univariate analysis showed that overexpression of NUDT10 was markedly correlated with poor overall patient survival in GC (hazard ratio [HR] = 1.064; 95% CI: 1.0012–1.118; *P*= 0.0156). We found that age, stage, and TNM classification were significantly associated with poor survival outcomes. As shown in Table 4 and Figure 4, multivariate Cox analysis of the clinicopathologic variables showed that high NUDT10 expression and age were independent risk factors for GC (HR = 1.089; 95% CI: 1.032–1.149, *P*= 0.0018 and HR = 1.042, 95% CI: 1.021–1.063, *P*< 0.001, respectively).

**Table 4.** Univariate and multivariate analyses of the correlation between NUDT10 and survival outcome in patients with gastric cancer (GC) in the TCGA cohort

	Univariate analysis			Multivariate analysis		
parameter	HR	95%CI	P-value	HR	95%CI	P-value
Age	1.027	1.008–1.046	0.006**	1.044	1.023–1.066	<0.001***
Gender	1.484	0.980–2.247	0.062	1.587	1.039–2.424	0.032*
Grade	1.368	0.947–1.977	0.095	1.381	0.936–2.037	0.103
Stage	1.535	1.221–1.931	<0.001***	1.244	0.807–1.918	0.323
T	1.298	1.023–1.645	0.031*	1.097	0.789–1.527	0.582
M	2.048	1.096–3.827	0.024*	2.375	1.060–5.325	0.036*
N	1.267	1.069–1.502	0.006**	1.146	0.892–1.472	0.287
NUDT10	1.639	1.116–2.406	0.011*	1.958	1.370–0.798	<0.001***

### NUDT10-related pathways by GSEA

**Figure 5.**
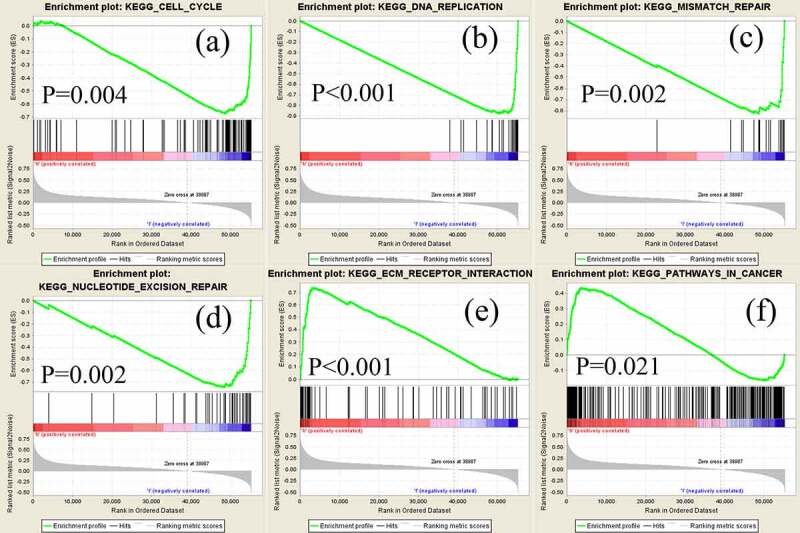
Significantly enriched signaling pathways revealed by gene set enrichment analysis. Genes involved in cell cycle (a), DNA replication (b), mismatch repair (c), nucleotide excision (d), extracellular matrix receptor (e), and pathways in cancer (f) were significantly enriched in NUDT10-related GC

GSEA was performed to screen for potential signaling pathways by comparing the high and low NUDT10 expression subgroups in the Molecular Signatures Database (MSigDB). Random sample permutations of 1000 were carried out, and significant gene set enrichment was defined with nominal *P*-value <0.05 and FDR q-value <0.05. As shown in Table 5 and Figure 5, based on normalized enrichment scores, we identified several significantly enriched signaling pathways, including cell cycle, DNA replication, mismatch repair, nucleotide excision repair, extracellular matrix (ECM) receptor integration, and cancer signaling (FDR <0.01), that were related to high expression of NUDT10 in GC.

**Table 5.** Enriched gastrocarcinoma-related gene sets

Gene set name (KEGG)	Size	NES	NOM p-val	FDR q-val
NUCLEOTIDE_EXCISION_REPAIR	44	−2.14	0.002	0.002
DNA_REPLICATION	36	−2.13	<0.001	0.001
CELL_CYCLE	124	−2.07	0.003	0.003
MISMATCH_REPAIR	23	−2.03	<0.001	0.005
ECM_RECEPTOR_INTERACTION	84	2.16	<0.001	0.001
PATHWAYS_IN_CANCER	325	1.64	0.021	0.100

Abbreviations: KEGG, Kyoto Encyclopedia of Genes and Genomes.

## Discussion

The human genome contains 24 NUDT hydrolase genes and at least five pseudogenes [21], but little is known about the role of NUDT genes in the field of oncology. NUDT family members are distinguished by the Nudix box, which is a 23-residue sequence motif that acts as a housecleaning enzyme [22,23]. The typical NUDT reaction produces substances such as phosphate, pyrophosphate, or N-methyl-2-pyrrolidone [24]. As a member of the NUDT family, NUDT10 has been reported to promote cell proliferation, suppress apoptosis, and trigger the loss of tumor suppressor genes [10], which suggest its role as a promoter of cancer development and progression. Our findings are consistent with those of previous studies.

The expression pattern of NUDT10 and its correlation with clinicopathological factors in GC remain unknown. The present study is the first to explore the role and clinical correlations of NUDT10 in GC. We demonstrate that NUDT10 is an independent prognostic factor for patient survival in GC. NUDT10 expression was significantly reduced in tumor tissues compared to normal tissues. High expression of NUDT10 in GC is significantly correlated with lymph node metastasis, TNM stage, and depth of local invasion. ROC curve analysis revealed a moderate diagnostic value for NUDT10 in GC.

We identified several cancer-related significantly enriched signaling pathways, mainly including ECM interaction and repair of genetic alteration, which are related to high NUDT10 expression in GC. The ECM is an important component of the tumor microenvironment and plays a key role in tumor progression and patient survival [25]. Zhou L, et al. constructed a novel prognostic signature for GC based on large sequencing data and showed that ECM-receptor interaction is an important platform for the function of prognosis-related differentially expressed genes [26]. Meanwhile, repair of genetic alterations is typically related to function, as shown by the GSEA results. According to previous studies, the function of NUDT might be related to reactive oxygen species and substances produced in the process of regular electron transport in cellular oxidative metabolic pathways, such as protein, lipid, and nucleic acid pathways. Reactive oxygen species cause functional or structural abnormalities in cells [27]. Oxidative damage to nucleic acids might induce a mismatch with nucleotides, leading to alterations in gene information. Accumulation of aberrant genomes can cause mutagenesis or cell death. Gene alterations can be altered through the functions of the NUDT family [28]. Thus, NUDT10 may have both oncogenic and tumor-suppressive functions in human cancer. Based on this, it can be inferred that the aberrant expression of NUDT10 attenuates DNA repair competence and increases genetic alteration, which is an essential step in tumorigenesis. This might explain why NUDT10 is expressed at low levels in GC tissues compared with normal tissues. Conversely, patients with GC who had higher NUDT10 had significantly worse prognosis than those with lower NUDT10. One possible explanation that can be extrapolated from our data is a null mutation, which leads to increased NUDT10 transcription and nonfunctional protein products. Under these conditions, although NUDT 10 is highly expressed in tumor tissues, it loses its function in correcting gene alteration, which is consistent with the poor prognosis in the higher NUDT10 subgroup.

The GSEA results showed that NUDT10 was mainly involved in genetic mutation repair and ECM receptor interaction. This result not only validates the characteristics of the NUDT family, but also reveals an underlying crosstalk between NUDT10 and the tumor microenvironment.

In summary, using bioinformatics analysis and our validating cohort, we analyzed the correlation between NUDT10 and the clinical factors and survival outcomes in GC. We firstly found that high expression of NUDT10 is correlated with advanced tumor stage, deeper local invasion, and worse survival outcomes in patients with GC. Nevertheless, there are also several limitations to this study. Firstly, due to the limitation of sample size, our validating result from HPA database is less convincing and more immunohistochemical validation of NUDT10 in gastric cancer are needed. Secondly, our transcriptome data is from TCGA database, combined analysis of multiple transcriptome profiling datasets may possibly provide better association analysis and survival analysis. Finally, as our study is based on bioinformatics analysis, our present study is unable to determine detailed biological mechanisms of NUDT10 in GC, further experimental exploration of NUDT10 is necessary.

## Conclusions

Based on bioinformatics analysis of several public databases and validating cohort, we demonstrated that high expression of NUDT10 represented a potential biomarker in GC. Furthermore, the ECM interaction and repair of genetic alteration may be key regulating pathways of NUDT10 expression in GC. More functional experiments and validation of NUDT10 in GC are worth exploring for further studies.

## Data Availability

The datasets or profiles used in the current study are from several databases, which are available at the following websites: TCGA database: https://portal.gdc.cancer.gov/repository HPA database: https://www.proteinatlas.org/ Kaplan-Meier Plotter database: https://kmplot.com/analysis/index.php?p=service&cancer=gastric

## Research highlights

- NUDT10 expression is significantly lower in GC tissues than in normal tissues
- High NUDT10 expression is correlated with advanced tumor stage and deeper invasion
- Extracellular matrix interaction and genetic alteration repair mainly are enriched in GC
